# A Kinetic Study of the Main Guaco Metabolites Using Syrup Formulation and the Identification of an Alternative Route of Coumarin Metabolism in Humans

**DOI:** 10.1371/journal.pone.0118922

**Published:** 2015-03-10

**Authors:** João Cleverson Gasparetto, Rosângela Gonçalves Peccinini, Thais Martins Guimarães de Francisco, Letícia Bonâncio Cerqueira, Francinete Ramos Campos, Roberto Pontarolo

**Affiliations:** 1 Department of Pharmacy, Universidade Federal do Paraná, Curitiba, PR, Brazil; 2 Department of Natural Active Principles and Toxicology, Universidade Estadual Paulista Julio de Mesquita Filho, Araraquara, SP, Brazil; Kobe University, JAPAN

## Abstract

For decades guaco species have been empirically used for the treatment of respiratory diseases. However, studies have shown that the toxic and therapeutic effects of the main guaco metabolites are dose-dependent, and none clinical study was done to evaluate the behavior of these substances in humans. In this work, a pilot study measuring the kinetic profile of the main guaco metabolites was performed leading to the knowledge of an alternative route of coumarin metabolism in humans. Initial screenings demonstrated that the administration of 60 mL of guaco syrup (single dose) did not provide sufficient levels of coumarin (COU), 7-hydroxycoumarin (7-HCOU), o-coumaric acid (OCA) and kaurenoic acid (KAU). The pharmacokinetic parameters were calculated by orally administering 60 mL of guaco syrup spiked with 1500 mg of COU. The kinetic study demonstrated that the plasmatic levels of 7-HCOU (considered the main metabolite of COU) were 10 times lower than the levels of COU, and the kinetic profile of 7-HCOU suggests sequential metabolism in the liver with low access of 7-HCOU to the systemic circulation. The study also demonstrated that OCA is one of the main bioavailable metabolites of COU. Therefore, the hydrolysis of the lactone ring forming a carboxylated compound is one of the possible routes of COU metabolism in humans. The half-lives of COU, 7-HCOU and OCA were approximately 4.0, 1.0 and 3.0 h, respectively and there was evidence that the recommended dosage of guaco syrup did not provide sufficient levels of COU, 7-HCOU or OCA to obtain a bronchodilation effect. Clinical studies are necessary to prove the efficacy and safety of products based on guaco.

## Introduction

*Mikania laevigata* and *M*. *glomerata*, commonly known as guaco, are important medicinal species widely used by the South American population for the treatment of respiratory diseases [1]. Guaco extracts have been reported to have anti-inflammatory, anti-allergy and bronchodilation properties [2,3,4,5], and due to their important effects, guaco syrup and other preparations are freely distributed through various government phytotherapy programs [6,7].

The pharmacological effects of guaco have been assigned to some of its major metabolites, such as coumarin, *o*-coumaric acid and kaurenoic acid. The *o*-coumaric acid (OCA) associated with coumarin reduced the number of leukocytes and eosinophils present in the lung tissue of mice in an induced allergic pneumonitis model. This finding suggested that both substances are parts of the phytocomplex responsible for the therapeutic activities of guaco species in allergic pneumonitis [8].

Kaurenoic acid (KAU) has demonstrated anti-inflammatory activity by dose-dependent inhibition of the synthesis of nitric oxide (IC_50_ = 51.73 μM), the release of prostaglandin E_2_ (IC_50_ = 106.09 μM) and the expression of cyclooxygenase-2 activity (IC_50_ = 3.5 μg/mL) in LPS-induced RAW264.7 macrophages. In a carrageenan-induced paw edema model in mice, KAU (50 mg/kg) reduced 34.4% of the swelling that occurred 5 h after the induction of inflammation [9]. At 10 μM and greater, KAU also had concentration-dependent activity on vascular smooth muscle (endothelium-intact or denuded rat aortic rings) that was pre-contracted with phenylephrine and potassium chloride [10,11].

Despite the therapeutic relevance of OCA and KAU, the benefits of guaco on the airways have been attributed mainly to the presence of simple coumarin (1,2 benzopyrone), which has been identified as the main bioactive component present in guaco extracts [12]. Coumarin (COU) has been reported to activate macrophages and the cells of the immune system and also to reduce acute and chronic protein edema [13,14]. COU also promoted *in vitro* a relaxing effect on guinea pig tracheas pre-contracted with histamine (EC_50_ = 35.0 μg/mL) or carbachol (EC_50_ = 33.4 μg/mL). *In vivo*, guinea pigs pre-treated with coumarin showed significant resistance to histamine inhalation, with an adequate dose of 75 mg/kg protecting 50% of the tested animals (AD_50_). These results suggest a significant bronchodilation effect of COU against chronic inflammatory disorders of the airways [15].

The use of COU has been reported for a long time, especially during the 1980 and 1990s, when COU was used as an adjuvant in melanoma therapy [16]. Clinical studies have demonstrated that in humans, COU is quickly converted during the first pass into 7-hydroxycoumarin (7-HCOU), the primary clinically active metabolite of COU *in vivo* [17]. This metabolite has also been described as an important agent for promoting significant attenuation of allergic airway inflammation in mice (60 mg/kg) [18].

As demonstrated, the presence of COU, 7-HCOU, OCA and KAU has been directly correlated with the benefits of guaco, but studies have shown that some of these compounds are toxic. For example, KAU has been shown to kill sea urchin embryos and to cause hemolysis in mouse and human erythrocytes [19]; it also induced DNA breaks, cytogenetic abnormalities in human peripheral blood leukocytes and positive genotoxic effects in the liver, kidneys and spleen of mice [20]. KAU has also shown dose-dependent genotoxicity in Chinese hamster lung fibroblast cells [21].

COU has been shown to be carcinogenic, especially in the liver and lungs of rats and mice [22]. With long exposure, this substance can change biochemical and hematological parameters and can cause ulcers and necrosis, fibrosis and cytologic alterations in the liver [23]. In humans, the majority of tests for mutagenicity and genotoxicity have suggested that COU is not toxic. This low toxicity has been attributed to the mechanism of the detoxification of COU, which in humans occurs via the 7-hydroxylation pathway. In rats and mice, the main route of detoxification is by 3,4-epoxidation, resulting in the formation of toxic metabolites [22].

Because the toxic and therapeutic effects of the main guaco metabolites are dose-dependent and the dosages of these compounds are not standardized in guaco phytomedicines [24,25], monitoring the kinetics of COU, 7-HCOU, OCA and KAU following guaco syrup administration has become important, especially to understand the half-life of these substances and to propose the correct posology of preparations without putting human health at risk.

The aim of this work was the conduction of a pilot study evaluating the kinetic profile of COU, 7-HCOU, OCA and KAU in human volunteers who orally received guaco syrup. The objective was to understand the kinetic characteristics of each compound for the establishment of a basis for future clinical studies that evaluate the genuine effectiveness of guaco syrup. An alternative route of coumarin metabolism in humans was also known through the conduction of the pharmacokinetic study.

## Experimental

### 1. Samples, chemicals and reagents

Blank, hemolyzed and lipemic plasmas were kindly donated by Centro de Hematologia e Hemoterapia do Paraná (Hemepar, Curitiba, Brazil). Samples of guaco syrup (same brand and lot) containing 0.295 mg/mL of COU, 0.018 mg/mL of OCA and 0.148 mg/mL of KAU, were obtained from local markets (Curitiba, Brazil). The amounts of each compound were determined by HPLC-MS/MS [24]. Methanol and acetonitrile (HPLC grade) were purchased from Tedia (Fairfield, USA), and formic acid (88%) was obtained from J. T. Baker Chemicals B.V. (Deventer, the Netherlands). Ammonium formate (97%) was purchased from Acros Organics (New Jersey, USA). Ultrapure water was obtained using a Milli-Q purification system from Millipore Corporation (Bedford, USA). *tert*-butyl methyl ether (99.8%) and standards of COU (99.0%), 7-HCOU (99.8%) and OCA (97.0%) and internal standards (IS) of 6-methylcoumarin (99.0%), isoferulic acid (98.0%) and prednisone (99.0%) were purchased from Sigma Aldrich (St. Louis, USA). KAU was isolated from *M*. *lanuginosa* DC, was purified, was identified (IR, GC/MS, 1H, and 13C NMR, S1 Fig.) [26], and was kindly donated by Dr. Obdulio Gomes Miguel of the Phytochemical Laboratory of Universidade Federal do Paraná.

### 2. Standard solutions

Stock solutions of COU, 7-HCOU, OCA, KAU and ISs were prepared separately in methanol at concentrations of 1 mg/mL. All the stock solutions were stored in amber bottles at 4°C. Working standard solutions were freshly prepared from these stock solutions as needed for each experiment, through appropriate dilution with acetonitrile/water (70:30 *v/v*).

### 3. Ethics statement, study design and blood collection

The pharmacokinetic study of COU, 7-HCOU, OCA and KAU was conducted in fourteen human volunteers (both sexes) with ages ranging from 22 to 35 years old. The weight ranged from 63 to 88 kg and the height from 1.65 to 1.80 m. The volunteers were submitted to the medical consultations and clinical analyses before their participation in the study. Only healthy volunteers (normal values of blood pressure and renal, hepatic, biochemical and hematological parameters) were eligible to participate in the study. The experimental protocols involving humans in this study were approved by the Ethics Committee of Universidade Federal do Paraná (CEP/SD 706041–005/CAAE 0018.0.091.00–09). The volunteers were informed about all the procedures and possible risks related to the study. All the volunteers provided written informed consent to participate in the study.

Initial screening of COU, 7-HCOU, OCA and KAU bioavailability was conducted in five volunteers (two female and three male) who orally received 60 mL of guaco syrup. A second screening was conducted in four volunteers (two male and two female) who orally received 60 mL of guaco syrup spiked with distinct amounts of pure COU. Each volunteer received only one of the following doses: 60 mL of guaco syrup containing 100, 500, 1000 or 1500 mg of COU. Final data for the pharmacokinetic parameters were obtained by oral administration to five volunteers (three male and two female) of 60 mL of guaco syrup spiked with 1500 mg of pure COU. None of the volunteers participated in more than one of the different studies and no adverse effect was observed during study conduction.

All the volunteers fasted for 8 h prior to the administration of guaco syrup and blood collection. Blood samples (3.0 mL) were collected (using flexible catheter 18–20G) before the syrup administration (zero min) and at fifteen time points after the syrup administration (10, 20, 30, 45, 60, 75, 90, 120, 150, 180, 240, 360, 480, 540 and 600 min). The anticoagulant used was tetrasodium edetate. All the samples were immediately centrifuged at 4000 rpm (5.0 min, room temperature), and the plasma was aliquoted and frozen at -40°C until analysis. The pharmacokinetic analysis was performed using a non-compartmental model, considering an extravascular administration route and using WinNonlin Professional software, version 5.3. The relationships among the areas under the curves (r areas = AUC_0-t_/AUC_0-inf_), were used to confirm the suitability of the experimental design. Values greater than 0.8 indicated an appropriate experimental design.

### 4. Plasma sample preparation

All the frozen (-40°C) human plasma samples from the volunteers were thawed at room temperature, and a 200 μL aliquot was transferred into 2 mL plastic centrifuge tubes. The analytes were extracted as follows.

#### 4.1. Extraction of COU and 7-HCOU using liquid-liquid extraction with *tert*-butyl methyl ether (TBME)

The 200 μL aliquots of thawed samples were spiked with 50 μL of acetonitrile/water (70:30 *v/v*) and 50 μL of the working IS solution to obtain a final concentration of 500 ng/mL of 6-methylcoumarin. The spiked samples were vortexed for 2 min, and a 1.5 mL aliquot of TBME was added to the tube. The samples were vortexed for 2 min and then submitted to centrifugation for 10 min (14000 rpm, -5°C). A 1.2 mL aliquot of each sample supernatant was transferred to a new plastic centrifuge tube, and TBME was evaporated at 40°C in a sample concentrator (CentriVap Labconco, Kansas City, EUA). The remaining samples were resuspended in 200 μL of acetonitrile/water (70:30 *v/v*) using a vortex mixer for 3 min.

#### 4.2. Extraction of OCA and KAU using protein precipitation with acetonitrile

The 200 μL aliquots of the thawed samples were spiked with 50 μL of acetonitrile/water (70:30 *v/v*) and 50 μL of the working IS solution to obtain a final concentration of 500 ng/mL of prednisone (IS) and 1000 ng/mL of isoferulic acid (IS). The spiked samples were vortexed for 2 min, and a 1.5 mL of acetonitrile was added to the tube. The samples were vortexed for 2 min and then were submitted to centrifugation for 10 min (14000 rpm, -5°C). Each sample supernatant was transferred to a new plastic centrifuge tube, and acetonitrile was evaporated in a sample concentrator (40°C). The samples were resuspended in 200 μL of acetonitrile/water (70:30 *v/v*) using a vortex mixer for 3 min.

### 5. HPLC-MS/MS instrumentation and conditions

The MS and MS/MS parameters were optimized by automatic MRM using individual working standard solutions of COU, 7-HCOU, OCA, KAU and IS. The two most intense fragment signals for each compound were obtained (most intense used for quantification and second most intense for peak qualification), except for KAU, for which a low signal intensity of the fragments was observed after MS/MS optimization. For KAU, only the molecular ion was monitored (*m/z* 301.1). The ESI source parameters were optimized by automatic flow-injection analysis.

HPLC-MS/MS analyses were performed using an Agilent 1200 HPLC System (Wilmington, USA), which consisted of a G1312B binary pump, G1379B degasser and G1316B column oven. The HPLC was connected to a CTC Sample Manager (Model 2777, Waters Corporation, Milford, USA) that was operated at room temperature. Acetonitrile and methanol were used as wash solvents. The HPLC system was coupled to an Applied Biosystems MDS Sciex API 3200 Triple Quadrupole Mass Spectrometer (Toronto, Canada), equipped with a Harvard 22 Dual Model syringe pump (Harvard Apparatus, South Natick, USA) and an electrospray ionization (ESI) source. The ESI source was operated in the positive ion mode to monitor COU, 7-HCOU and 6-methylcoumarin (IS) and in the negative ion mode to monitor OCA, KAU, isoferulic acid (IS of OCA) and prednisone (IS of KAU). For the positive ion mode, the mobile phase consisted of acetonitrile/water/formic acid (70:30:0.05 *v/v/v*) at an elution rate of 200 μL/min in an isocratic system. For the negative ion mode, the mobile phase consisted of a gradient of water (A) and acetonitrile/water 95:5 *v/v* (B), both containing 1 mM ammonium formate. The flow rate was maintained at 400 μL/min, and the gradient profile was as follows: t_0–1.0min_: A = 70%; t_1.0–1.1min_: A = 5%; t_1.1–5.0min_: A = 5%; t_5.0–5.1min_: A = 70%; and t_5.1–9.0min_: A = 70%. For both methods, the analyte separations were achieved on an XBridge Shield RP18 150 x 2.1-mm (5-μm particle size) column coupled with an XBridge RP18 10 x 2.1-mm (5-μm particle size) guard column (Waters Corporation, Mildford, USA). The injection volume was 20 μL, and the column temperature was maintained at 25°C for positive ion mode and at 35°C for negative ion mode. Data acquisition was performed with the MS Workstation by Analyst software, version 1.4.2 (ABSciex). Quantification was performed in Multiple Reaction Monitoring (MRM) mode, maintaining the dwell time at 300 and 400 ms for the positive and negative ion modes, respectively. The ion transitions and the individual compound parameters are shown in Table 1. The ion-source parameters for ESI-positive mode were the following: curtain gas (CUR), 10 psi; collision gas (CAD), 6 psi; ion spray voltage (ISV), 5500 V; nebulizer gas (GS1), 40 psi; turbo gas (GS2), 40 psi; and temperature, 450°C. The ion source parameters for ESI negative mode were as follows: CUR, 18 psi; CAD, 6 psi; IS, -4500 V; GS1, 50 psi; GS2, 50 psi; and temperature, 550°C. The high-purity nitrogen and zero grade air that were used as the CUR, GS1, GS2 and CAD gases were produced using a high-purity nitrogen generator from PEAK Scientific Instruments (Chicago, USA).

**Table 1:** Compound-dependent parameters and ion transitions of the analytes and internal standards (ISs) used for the quantification.

Ionization mode	Compound	Molecular íon (*m/z*)	Ion transition (*m/z*)	CE^a^	CEP^b^	CXP^c^	DP^d^	EP^e^
				(V)	(V)	(V)	(V)	(V)
Positive [M+H]^+^	Coumarin	147.3	147.3 → 91.0	31	12	4	36	8
			147.3 → 103.1	25	8.4	4	36	8
	7-hydroxycoumarin	163.1	163.1 → 107.1	33	10.8	4	46	4
			163.1 → 77.0	43	11.5	4	46	10
	6-methylcoumarin (IS)	161.0	161.0 → 105.2	27	10	4	61	11.5
			161.0 → 77.1	45	10	4	61	11.5
Negative [M-H]^-^	*o*-coumaric acid	162.9	162.9 → 119.0	-18	-10	0.0	-25	-5
			162.9 → 93.1	-30	-10	0.0	-25	-5
	Kaurenoic acid	301.0	301.0	-10	-22	-4	-70	-5
	Isoferulic acid (IS)	192.8	192.8 → 134.2	-20	-10	-4	-25	-6.5
			192.8 → 133.5	-34	-10	-4	-25	-6.5
	Prednisone (IS)	357.0	357.0 → 327.0	-15	-18	-4	-25	-6.5
			357.0 → 299.0	-15	-18	-4	-25	-6.5

Data:

^a^CE, collision energy;

^b^CEP, collision cell entrance potential;

^c^CXP, cell exit potential;

^d^DP, declustering potential;

^e^EP, entrance potential.

### 6. Validation of the analytical methods

The proposed methods were validated as described in the Food and Drug Administration Bioanalytical Method Validation guidelines [27].

To evaluate the limits of detection (LOD) and lower limit of quantification (LLOQ), a 200 μL aliquot of blank plasma were spiked with 100 μL of working standard solutions to obtain 100 ng/mL of each compound. The samples were vortexed (2 min), submitted to extraction procedure and diluted in series (acetonitrile/water, 70:30 *v/v*) until the smallest detectable peak was obtained. The LOD was estimated at a signal-to-noise ratio of 3:1, and the LLOQ was estimated at a signal-to-noise ratio of at least 10:1, until the desired precision was obtained (RSD < 20%).

The selectivity was investigated by comparing the HPLC-MS/MS chromatograms of blank, hemolyzed and lipemic plasmas samples (from different lots) with blank plasma spiked with the analytes at LLOQ concentration of each analyte. No significant interfering peaks should have been observed at the retention times of the analytes and internal standards.

The linearity study was performed using the internal standardization method at nine concentration levels for each compound. Calibration curves were prepared in triplicate on five different days by spiking 200 μL of blank plasma with 50 μL of IS solution and 50 μL of analyte solution to obtain the following concentrations: positive ion mode: 10, 50, 100, 250, 500, 750, 1000, 1250 and 1500 ng mL of COU and 7.5, 12.5, 25, 50, 100, 250, 500, 750 and 1000 ng/mL of 7-HCOU, each level containing 500 ng/mL of 6-methylcoumarin (IS); negative ion mode: 10, 25, 50, 75, 100, 250, 500, 750 and 1000 ng/mL of OCA and 5, 10, 25, 50, 75, 100, 250, 500 and 750 ng/mL of KAU, each level containing 1000 ng/mL of isoferulic acid (IS) and 500 ng/mL of prednisone (IS). The slope, intercept and regression coefficient were calculated as regression parameters by weighted (1/x) linear regression. Variation less than 15% in accuracy and the precision at each level should have been expected, except with LLOQ, for which no variations greater than 20% should have been observed. The linear correlation coefficient (r) also must have been equal to or greater than 0.98.

Quality control (QC) samples were prepared in eight replication by spiking 200 μL of blank plasma with 50 μL of IS solution and 50 μL of analyte solution, to obtain four level concentrations each analyte, as shown in Table 2.

**Table 2:** Quality control levels used to evaluate the validation parameters and method performance.

	Compounds	Quality control levels	Concentration (ng/mL)
**Positive ion mode**	Coumarin	LLOQ	10.0
		LQC	50.0
		MQC	500.0
		HQC	1250.0
	7-hydroxycoumarin	LLOQ	7.5
		LQC	12.5
		MQC	100.0
		HQC	750.0
	6-methylcoumarin (IS)	-	500.0
**Negative ion mode**	*o*-coumaric acid	LLOQ	10.0
		LQC	25.0
		MQC	100.0
		HQC	750.0
	Kaurenoic acid	LLOQ	5.0
		LQC	10.0
		MQC	75.0
		HQC	500.0
	Isoferulic acid (IS)	-	1000.0
	Prednisone (IS)	-	500.0

Data: LLOQ: lower limit of quantification; LQC: low quality control; MQC: medium quality control; HQC: high quality control; IS: internal standard

To perform the carryover test, triplicates of QC samples at higher concentration levels (HQC) were prepared and injected into the HPLC-MS/MS alternately with blank plasma. No significant interfering peaks should have been observed in the blank plasma chromatogram at the retention times of the analytes and ISs.

Intra- and inter-day accuracy and precision were demonstrated by preparing QC samples in eight replications at four concentration levels (LLOQ, LQC, MQC and HQC) on three consecutive days. Intra- and inter-day precision was estimated by the residual standard deviation (RSD%) at each concentration level, while the accuracy was estimated by calculating the difference between the calculated and theoretical concentrations (relative error, RE%). For both accuracy and precision, variation must have been within 15%, except with LLOQ, for which the value of the variation should not have deviated by more than 20%.

The dilution test was performed in eight replication by spiking blank plasma with standard solutions to obtain a final concentration of 1600 ng/mL of COU, 850 ng/mL of KAU and 1100 ng/mL of 7-HCOU and OCA (original concentrations). The samples were diluted tenfold with blank plasma and were processed and analyzed. The results obtained with the diluted samples were compared with the original concentrations by RE%. The precision (RDS%) among the diluted replicates was also evaluated.

To evaluate the matrix effect, aliquots of 200 μL of normal, lipemic and hemolyzed plasmas from different lots were initially spiked with 100 μL of acetonitrile/water (70:30 *v/v*). The samples were vortexed (2 min) and submitted to the extraction procedures. The samples were resuspended in 100 μL of acetonitrile/water (70:30 *v/v*), 50 μL of IS solution and 50 μL of analyte solution to obtain the same concentration levels of the LQC and HQC samples. For each concentration level, the normalized effect of matrix (NEM) of the responses of the analytes in plasma and in solution was calculated (analyte response in matrix/IS response in matrix *versus* the analyte response in solution/IS response in solution [28]. Variations less than 15% relative to the NEM calculated to the all concentrations levels each analyte indicated that the matrix effect was not significant.

The stability of COU, 7-HCOU, OCA, KAU and ISs was tested using eight replications under a variety of storage and handling conditions. The short-term stability assay was performed by spiking 200 μL of blank plasma with 50 μL of analyte solution to obtain 50 and 1250 ng/mL for COU; 12.5 and 750 ng/mL for 7-HCOU; 25 and 750 ng/mL for OCA; and 10 and 500 ng/mL for KAU. The samples were vortexed for 2 min and then were kept at room temperature for 8 h. Afterward, the samples were spiked with 50 μL of IS solution and were vortexed (2 min) and submitted to the extraction procedures. The short-term stability was evaluated by comparing the mean recovery of the analytes and the IS obtained from stored samples with the mean values obtained with freshly prepared samples, at same concentration levels.

Freeze-thawed, post-preparative and long-term stability assay samples were prepared and evaluated similar to the short-term stability samples. However, the freeze thawing assay was assessed after undergoing three freeze-thaw cycles (one freeze-thaw cycle per day on three consecutive days) and post-preparative assay, maintaining the extracted samples for 24 hours in the Sample Manager (20°C, transparent vials). The long-term stability of human plasma stored at -40°C was assessed at the end of the study (60 days).

The stability of the stock solutions (1 mg/mL of each analyte and ISs) was evaluated after 30 days of storage in a refrigerator (4°C, amber bottles). The stored solutions were diluted with acetonitrile/water (70:30 *v/v*) to obtain the same concentration levels as the short-term stability assay. The stability of the stock solutions was evaluated by comparing the mean recovery of the analytes and IS obtained from stored samples to the mean values obtained with freshly prepared samples, at the same concentration levels. The stability of the working standard solutions was assessed using a procedure similar to that described for stock solution stability. However, the samples were stored for 6 h at room temperature.

### 7. Tests for sample clean-up

To achieve the best recovery of analytes and ISs, several procedures for sample clean-up were tested, including solid-phase extraction (SPE), protein precipitation with acetonitrile, and liquid-liquid extraction with *tert*-butyl methyl ether, chloroform, ethyl acetate and ether/hexane at a 70:30 *v/v* ratio. SPE was conducted using an Oasis HLB cartridge (Waters Corporation, Mildford, USA) previously conditioned with 1 mL of methanol and 1 mL of ultrapure water. After sample loading, the sorbent was washed with 1 mL of ultrapure water being the analytes eluted with 1 mL of acetonitrile. Protein precipitation and liquid-liquid extraction were conducted as previously described in Section Plasma Sample Preparation. All the extraction procedures were performed by preparing QC samples in eight replications at the MQC concentration level. The choice of the best procedure was based on the best recovery of analytes and IS and data reproducibility.

## Results and Discussion

### 1. Method development

To develop the HPLC-MS/MS methods, several combinations of methanol, water and acetonitrile were tested for mobile phase composition, maintaining 0.05% formic acid or 1 mM ammonium as an additive under all conditions. The flow rate (200 to 400 μL/min) and the column oven temperature (25–40°C) were also varied to achieve the best run time and peak shape. Considering all the tested conditions, acetonitrile provided better ionization of the analytes than methanol. Acetonitrile also reduced system pressure and no tailed peak or baseline noise was observed using acetonitrile as organic modifier in the mobile-phase composition.

**Fig 1:**
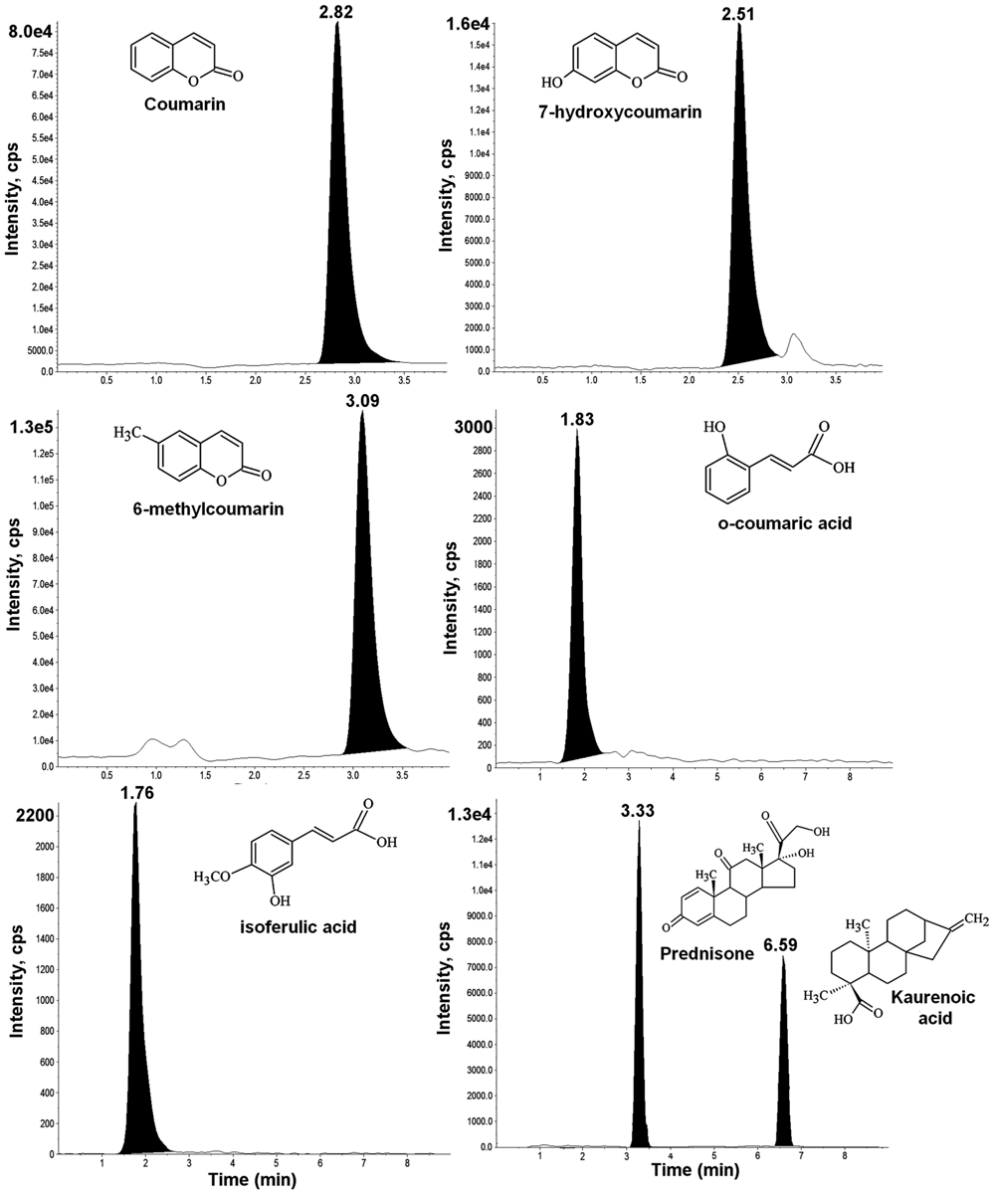
Representative HPLC-MS/MS chromatograms of human plasma spiked with standards of coumarin, 7-hydroxycoumarin, *o*-coumaric acid, kaurenoic acid and the internal standards 6-methylcoumarin, isoferulic acid and prednisone. Data: signals not smoothed; sample prepared at MQC concentration level.

The HPLC-MS/MS method for the determination of OCA and KAU is the unique developed in plasma matrix. The method developed for the determination of COU and 7-HCOU offered several advantages over other methods available in the literature. For example, higher sensitivity compared to HPLC-DAD [29,30,31,32,33], with a linear range of detection 100 times lower achieved with acceptable accuracy and precision. The new methods showed excellent peak shape and a short run time. No significant environmental waste was produced because the new methods required low flow rates and small amounts of organic solvent. The new methods were also fully validated according to current worldwide regulations, thus ensuring reliable results. The representative chromatograms for both methods are shown in Fig. 1.

### 2. Tests for sample clean-up

After method development, sample preparation was the most critical step in the analysis of COU, 7-HCOU, OCA and KAU in human plasma. Thus, several procedures for extraction were tested.

Solid phase extraction (SPE) showed high reproducibility for extracting COU and 7-HCOU. However, the presence of endogenous interference was observed at the retention time of the IS 6-methylcoumarin (data not shown), thereby compromising the assay. SPE was also not efficient for extracting OCA and ISOF, and high variations in KAU and PRED extraction were observed using SPE (RSD > 30%).

Protein precipitation (PPT) was the only efficient technique for extracting OCA, ISOF, KAU and PRED with high reproducibility (RSD < 7.5%). To extract COU, 7-HCOU and 6-MC, liquid-liquid extraction with TBME demonstrated higher efficiency and reproducibility than the other solvents (RSD < 4.5%). The mean individual extraction recoveries of the analytes and ISs using liquid-liquid extraction with TBME and protein precipitation were: COU (96.73 ± 1.97%), 7-HCOU (81.48 ± 2.15), 6-MC (96.05 ± 2.19), OCA (72.57 ± 3.41), KAU (88.59 ± 1.36), ISOF (91.28 ± 2.44) and PRED (97.11 ± 1.47). Liquid-liquid extraction with TBME was therefore considered effective for the extraction of COU and 7-HCOU from human plasma, and protein precipitation with acetonitrile effective for extraction of OCA and KAU.

### 3. Method validation

#### 3.1. Selectivity

The present methods did not reveal additional endogenous interference peaks at the retention times of the analytes and ISs. The developed methods were therefore considered selective. The chromatograms obtained with normal, hemolyzed, lipemic and spiked plasmas are presented in S2 and S3 Figs.

#### 3.2. Limit of detection (LOD), lower limit of quantification (LLOQ) and linearity

The high sensitivity of the developed methods was demonstrated by the low LOD (signal-to-noise ≥ 3) estimated at 1.5 ng/mL for KAU, 2.5 ng/mL for 7-HCOU, 2.5 ng/mL for OCA and 3.0 ng/mL for COU. The LLOQ, based on a signal-to-noise ratio ≥ 10 with appropriate precision (RSD <10%) was estimated at 5.0 ng/mL for KAU, 7.5 ng/mL for 7-HCOU and 10.0 ng/mL for OCA and COU.

All calibration curves showed excellent linearity with correlation coefficients (r) > 0.997. The means of the individual linear equations and correlation coefficients were as follows: COU, y = 0.918 *x* + 0.00186 (r = 0.9992); 7-HCOU: y = 0.739 *x* + -0.0032 (r = 0.9992); OCA, y = 4.71 *x* + 0.259 (r = 0.9973); and KAU, y = 2.77 *x* + 0.011 (r = 0.9991). At all the concentration levels, the variations in precision (RSD %) were less than 10%, with values ranging from 2.2 to 5.2% for COU; from 0.8 to 8.5% for 7-HCOU, from 0.53 to 9.6% for OCA, and from 2.5 to 9.8% for KAU. The individual values for accuracy at each concentration level ranged from 97.02 to 105.89% for COU, from 93.33 to 106.41% for 7-HCOU, from 95.83 to 107.50% for OCA and from 89.95 to 107.69% for KAU. These results guaranteed a reliable response, independent of the concentrations utilized.

#### 3.3. Precision, accuracy and sample dilution

The results for the accuracy and precision of the analyses are shown in Table 3. The new methods were precise for all the analytes and ISs, with RSDs varying from 2.72 to 9.96% for intra-day and from 1.97 to 10.14% for inter-day analysis. The present methods also showed satisfactory accuracy, with relative errors ranging from-10.09 to 9.88% for intra-day and from-7.56 to 2.66% for inter-day analysis. The analyses also demonstrated that samples subjected to dilution had the same precision and accuracy as undiluted samples (Table 3). Therefore, the developed methods were considered to be reproducible and accurate.

**Table 3:** Precision and accuracy of coumarin (COU), 7-hydroxycoumarin (7-HCOU), 6-methylcoumarin (6-MC), *o*-coumaric acid (OCA), kaurenoic acid (KAU), isoferulic acid (ISOF) and prednisone (PRED) obtained in human plasma by HPLC-MS/MS experiments.

	Compounds	Quality Control level	Accuracy			Precision	
			Standard concentration(ng/mL)	Intra-day RE%	Inter-day RE%	Intra-day (RSD%)	Inter-day (RSD%)
**Positive ion mode**	COU	LLOQ	10.0	-10.09	-7.56	5.91	5.05
		LQC	50.0	-1.71	1.15	2.72	2.98
		MQC	500.0	-2.44	2.66	2.75	2.93
		HQC	1250.0	-7.16	-4.72	3.91	3.93
		DQC	1600.0	-1.66	-1.84	2.90	1.97
	7-HCOU	LLOQ	7.5	-1.03	-0.88	9.95	8.47
		LQC	12.5	9.88	2.57	4.34	6.76
		MQC	100.0	-3.82	0.92	3.50	4.37
		HQC	750.0	-1.37	-1.53	3.56	5.19
		DQC	1100.0	-1.44	0.35	3.31	4.19
	6-MC*	-	500.0	5.31	0.96	3.66	6.09
**Negative ion mode**	OCA	LLOQ	10.0	0.29	-3.56	8.96	10.14
		LQC	25.0	-3.89	-0.27	5.64	4.77
		MQC	100.0	-2.56	1.02	3.99	3.86
		HQC	750.0	-1.98	-0.81	4.17	2.91
		DQC	1100.0	2.11	-0.43	3.38	4.82
	KAU	LLOQ	5.0	-8.15	-3.51	9.96	8.79
		LQC	10.0	-3.64	-3.87	4.14	6.07
		MQC	75.0	-1.60	-2.01	3.27	2.89
		HQC	500.0	2.26	1.55	4.44	3.75
		DQC	850.0	-2.79	-2.12	2.97	3.07
	ISOF*	-	1000.0	-0.81	1.94	2.87	3.66
	PRED*	-	500.0	0.76	0.30	3.22	3.94

Data: LLOQ: lower limit of quantification; LQC: low quality control; MQC: medium quality control; HQC: high quality control; DQC: dilution quality control; Intra-day analysis, n = 8; Inter-day analysis, n = 24; RE%: relative error; RSD%: relative standard deviation; *internal standard

#### 3.4. Matrix effect and carryover test

As demonstrated in Table 4, the variations of the normalized effects of matrix (NEM) of each compound were less than 15%, indicating that the effects of the biological matrix on the response of the analytes and ISs were insignificant. With alternate injections of QC (highest evaluated level) and blank plasma samples, no significant interfering peaks were observed at the retention times of the analytes and ISs. There was therefore no risk of carryover contamination between the injections.

**Table 4:** Variation in the normalized effects of the matrix (NEM) of coumarin, 7-hydroxycoumarin, *o*-coumaric acid and kaurenoic acid, calculated to assess matrix effect (n = 8).

Compounds	Concentration (ng/mL)	NEM	Mean ± S.D.	NEM (RSD%)
Coumarin	50.0	0.87 ± 0.06	0.94 ± 0.09	9.23
	1250.0	1.01 ± 0.05		
7-hydroxycoumarin	12.5	1.02 ± 0.05	1.02 ± 0.04	4.18
	750.0	1.03 ± 0.03		
*o*-coumaric acid	25.0	0.95 ± 0.09	1.03 ± 0.11	10.77
	750.0	1.11 ± 0.08		
Kaurenoic acid	10.0	1.19 ± 0.10	1.10 ± 0.15	13.62
	500.0	1.00 ± 0.13		

#### 3.5. Stability assay

S1 and S2 Tables summarize the mean recovery of the analytes and ISs obtained from the stored samples and their comparisons with the mean values obtained from freshly prepared samples at the same concentration levels. Under the conditions evaluated, an RSD and RE < 15% demonstrated no significant differences between the amounts obtained with freshly samples and those obtained after a variety of storage and handling conditions. Excellent stability for all the compounds was therefore shown.

### 4. Pharmacokinetic study

The two developed and validated HPLC-MS/MS methods were applied to a kinetic study of COU, 7-HCOU, OCA and KAU. In volunteers who receiving an oral dose of 60 mL of guaco syrup (17.6 mg of COU, 1.1 mg of OCA and 8.9 mg of KAU), no peak absorption of the substances was observed until 600 min after syrup administration. The administration of guaco syrup therefore did not provide sufficient levels of COU, 7-HCOU, OCA and KAU to be detected by sensitivity methods, even using a dose of guaco syrup 12 times greater than that recommended in the packaging of the phytomedicines (5 mL).

The low bioavailability demonstrated in the initial screening led to the conclusion that the kinetic study of COU, 7-HCOU, OCA and KAU was possible only by spiking guaco syrup with known amounts of these substances. However, kaurenoic acid has been shown to be toxic [16,19,20,21], and the potential for toxicity of *o*-coumaric acid is still unknown. The administration of guaco syrup spiked with 7-HCOU makes no sense because 7-HCOU is not a metabolite of guaco, but it originates from the hepatic metabolism of COU [17].

The administration of guaco syrup spiked with COU is practicable because COU is not toxic to humans. COU is also the main component of guaco species, and its use has been reported for many years for the treatment of various clinical conditions [17]. However, there is no consensus regarding the correct dosage of COU to conduct a kinetic study, and data from literature have demonstrated that the amounts of intake of COU can vary substantially according to the therapy or study (e.g., 30 to 3000 mg) [14,32,34,35].

A second screening was therefore necessary to evaluate the plasmatic response of COU based on different doses of the active. The dose of COU that promoted the bronchodilator effect in guinea pigs (AD_50_: 75 mg/kg) [15] was used as a reference to conduct the study. Using allometric extrapolation [36,37], the dose to obtain a bronchodilator effect in humans was calculated, resulting in 21 mg/kg (1470.0 mg of COU for a 70 kg individual).

**Fig 2:**
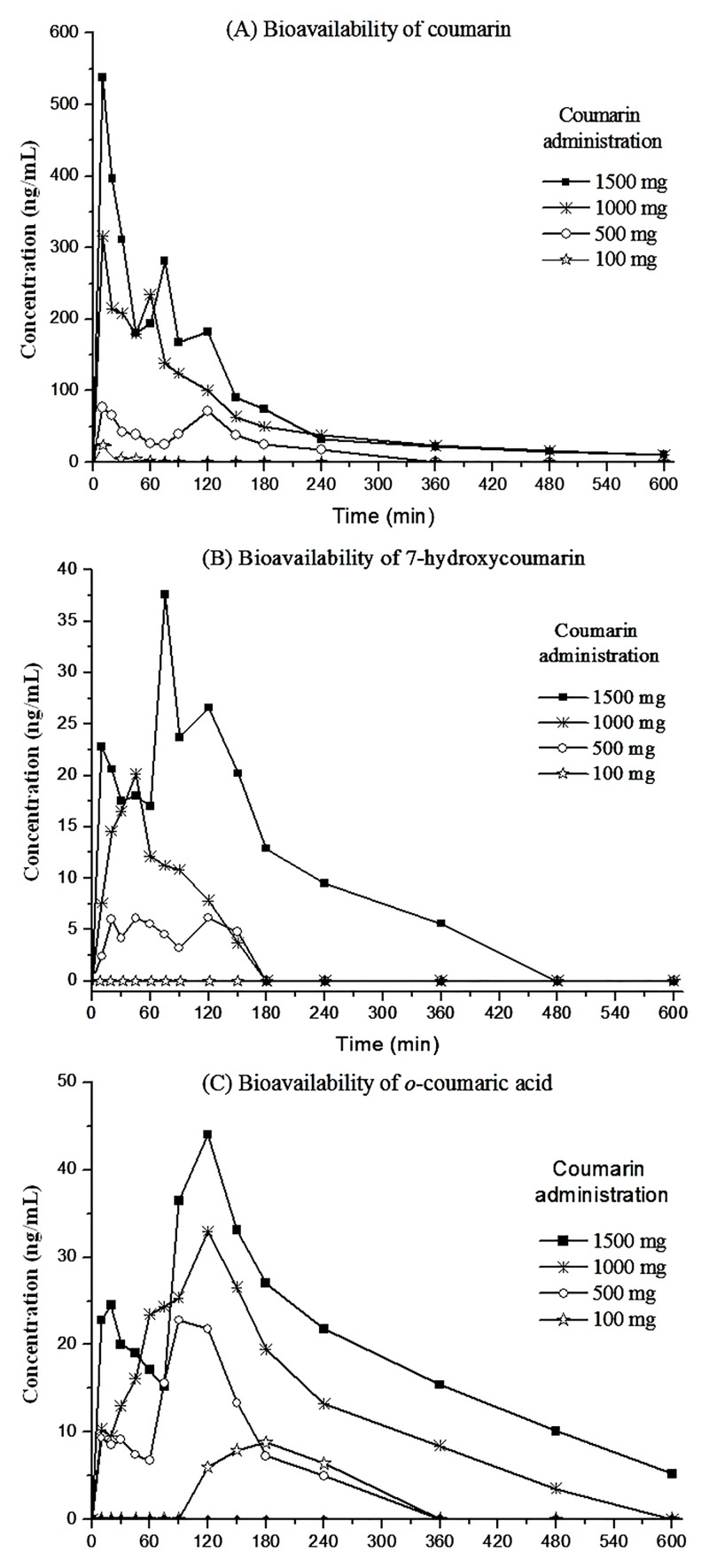
Temporal profile of (A) coumarin, (B) 7-hydroxicoumarin and (C) *o*-coumaric acid plasma concentrations in human volunteers receiving oral administration of 60 mL of guaco syrup spiked with different amounts of coumarin.

Although COU has been described as a low-toxicity substance in humans, the possibility of the occurrence of side/toxic effects should be considered during study conduction. Progressive amounts of COU were thus selected to spike guaco syrup, aiming for the possibility of study conduction using smaller doses than those allometrically extrapolated (1470.0 mg). The screening was performed using four volunteers who orally received 60 mL of guaco syrup spiked with 100, 500, 1000 and 1500 mg of pure COU (each volunteer receiving one dose). As a result, peak absorption of COU was observed in all four volunteers 10 min after syrup administration (Fig. 2A), indicating that COU was quickly absorbed from the gastrointestinal tract, most likely due to its non-polar and high partition coefficient (log K_o/w_: 1.39) characteristics.

Following peak absorption (10 min), the rapid decline in available COU suggested immediate distribution throughout the body. The peak concentration in plasma appeared within 45 to 120 min, with rapid elimination clearly exhibited 180 min after syrup administration (Fig. 2A). At a 100 mg dosage, quantifiable levels of available COU were determined at only 10 min after syrup administration (22.1 ng/mL). At other time points, only traces or none of available COU was detected in the plasma volunteers (Fig. 2A).

The dosages of 500 and 1000 mg of COU were ideal to determine the main pharmacokinetic parameters because an adequate temporal profile was obtained. However, at these dosages, the amounts of converted 7-HCOU were insufficient to construct an adequate temporal profile of the main metabolite of COU (Fig. 2B). At only 1500 mg of COU, a reliable temporal profile of 7-HCOU was obtained, making possible the calculation of the main pharmacokinetic parameters of 7-HCOU (Fig. 2B). The screening also demonstrated an unexpected presence of OCA in plasma volunteers who orally received guaco syrup spiked with different concentrations of COU (Fig. 2C). Therefore, another route of COU metabolism in humans was evidenced.

Another relevant point clearly evidenced was the existence of a direct relationship between the amounts of available COU and the amounts of OCA and 7-HCOU in plasma volunteers (Fig. 2). In summary, if the biological effects of OCA and 7-HCOU exist, they will exist dependently on the COU dosage.

**Fig 3:**
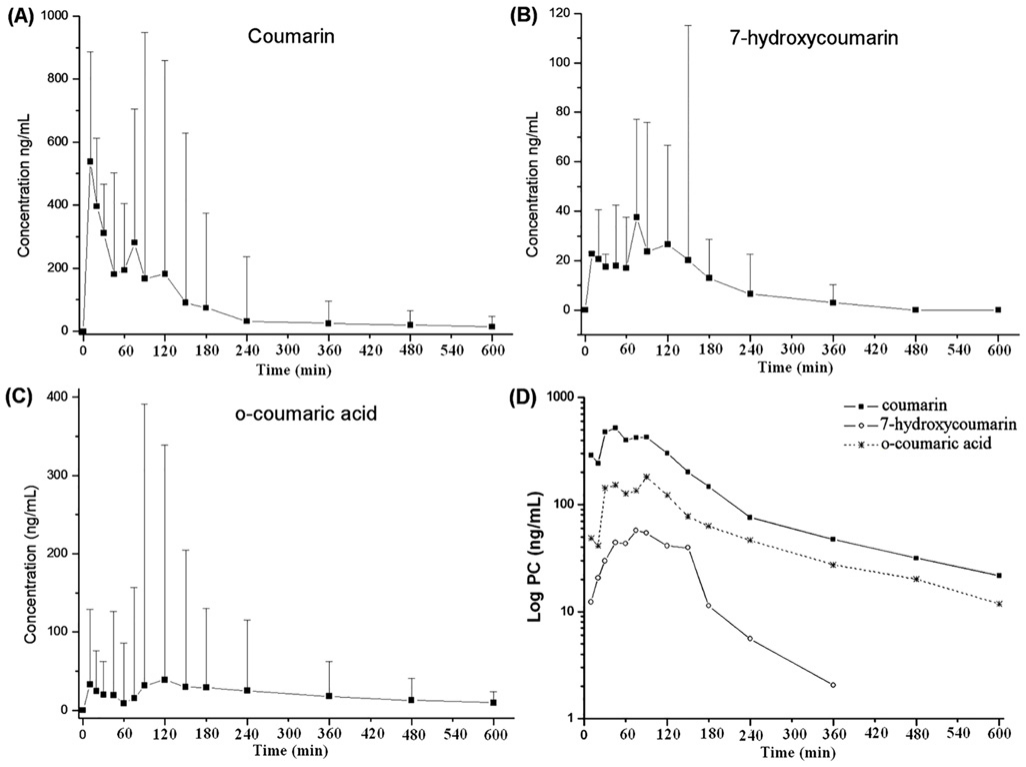
Temporal profile of the mean plasma concentrations of coumarin, 7-hydroxycoumarin and o-coumaric acid in human volunteers receiving oral administration of 60 mL of guaco syrup spiked with 1500 mg of coumarin.

From the results obtained with the second screening, the bioavailability study of COU, 7-HCOU and OCA was concluded in a third study, which included the participation of five volunteers who orally received 60 mL of guaco syrup spiked with 1500 mg of COU (dose allometric extrapolated). At this dosage, quantifiable levels of COU, 7-HCOU and OCA were successfully obtained up to 600 min after syrup administration, and the main pharmacokinetic parameters of each compound were successfully determined. The mean plasma concentration (ng/mL) and deviations of COU, 7-HCOU and OCA obtained at all-time points of blood collection are show in Fig. 3A, B and C and the Log of plasma drug concentration versus time curve is shown in Fig. 3D.

The individual temporal profiles of the mean plasma concentrations of COU, 7-HCOU and OCA demonstrated the presence of three peaks of absorption (Fig. 3D). COU and OCA presented peak plasma concentrations at the time points of 10, 45 and 90 min, while 7-HCOU presented peak concentrations at 45, 75 and 120 min. These data suggests the possibility of enterohepatic circulation of COU and metabolites.

The occurrence of enterohepatic circulation for COU and metabolites is contradictory to the conclusions described by Shilling and co-authors [38], who claimed that the possibility of biliary excretion for COU was insignificant due to extensive first-pass metabolism and rapid elimination from the body. In our case, the enterohepatic circulation must be associated with the dosage of COU, which was 7.5 times greater than the dosage used by Shilling and co-authors (200 mg) [38]. Using higher dosage, the expectation is the saturation of COU converting enzymes (CYP2A6 and UDP-glucuronyltransferase).

The saturation of the cytochrome/converting enzymes also justifies the plateau observed in the kinetic curve of each substance (Fig. 3D). Note in Fig. 3D that the plasma concentration levels were maintained while the mechanisms of drug metabolism were saturated. At the end of saturation, the phase of elimination became the main process.

Considerable differences in bioavailability among COU, 7-HCOU and OCA were also observed through the individual temporal profiles of each substance (Fig. 3D). The amounts of available 7-HCOU were approximately 10 times less than the amounts observed in the overall temporal profile of COU. These data were confirmed by the calculated areas under the curves (AUC_0-t_) of 1341.0 for COU and 126.0 for 7-HCOU (Table 5). The individual temporal profile of OCA demonstrated an AUC_0-t_ of 465.0, which was approximately three times greater than that calculated for 7-HCOU. This inedited result demonstrated that OCA, more than 7-HCOU, is one of the main bioavailable metabolites of COU.

**Table 5:** Main pharmacokinetic parameters for coumarin (COU), 7-hydroxycoumarin (7-HCOU) and *o*-coumaric acid (OCA) in human plasma after oral administration of 60 mL of guaco syrup spiked with 1500 mg of coumarin (n = 5).

Parameters	COU	7-HCOU	OCA
	(Mean ± S.D.)	(Mean ± S.D.)	(Mean ± S.D.)
C_max_ (ng/mL)	735.46 ± 252.29	84.98 ± 40.15	230.64 ± 186.98
T_max_ (h)	0.93 ± 0.57	1.40 ± 0.68	1.45 ± 0.84
AUC_0–t_ (ng*h/mL)	1341.0 ± 515.0	126.0 ± 54.0^a^	465.0 ± 299.0^a^ ^,^ ^b^
AUC_0–∞_ (ng*h/mL)	1458.0 ± 547.0	134.0 ± 58.0^a^	518.0 ± 299.0^a^ ^,^ ^b^
r areas	0.92 ± 0.03	0.95 ± 0.05	0.87 ± 0.08
T_1/2_ (h)	4.01 ± 0.85	1.16 ± 0.82^a^	3.50 ± 0.83^b^
K_el_ (L/h)	0.18 ± 0.04	0.91 ± 0.59^a^	0.21 ± 0.06^b^
Vd/f (L)	6733.0 ± 3284.0	-	-
Cl/f (L/h)	1148.0 ± 411.0	-	-

Data: C_max_: maximum concentration of drug in human plasma observed at a particular time point; T_max_: time at which maximum concentration of drug in plasma was observed; AUC_0–t_: area under plasma concentration—time curve; AUC_0–∞_: area under plasma concentration curve extrapolated to infinity; T_1/2_: time required for decomposition of the drug to half of the concentration; K_el_: elimination rate constant; Vd/f: apparent volume of distribution; Cl/f: apparent rate at which a substance is removed or cleared from the body;

^a^significantly different from coumarin;

^b^significantly different from 7-HCOU (Student t test, 95% confidence).

The values of r areas greater than 0.8 confirmed that the experimental design was appropriate for calculating the pharmacokinetic parameters (Table 5). The statistical comparison (Student t test, 95% confidence, Prob>|t| = 0.04545) of the elimination rate constant (K_el_) demonstrated that 7-HCOU was more rapidly eliminated than COU (Table 5). The elimination rate constant of OCA did not demonstrate significant differences when compared with the K_el_ of COU.

That the K_el_ of OCA (0.21 L/h) was graphically equal to the K_el_ of COU (0.18 L/h) does not reflect the real rate of elimination of OCA. The rate of elimination of OCA should have been greater than the calculated value, because OCA is more polar than COU, and its characteristics facilitate its elimination. Additionally, when the elimination rate of the metabolite is superior to the elimination rate of its precursor, the terminal slope of the curve of the metabolite is graphically similar to the slope of the precursor drug (Fig. 3D), but the area under the curve is smaller (Table 5) [39]. This finding occurs because at the same time that the metabolite is formed, its excretion readily occurs, and body accumulation does not happen. The elimination of OCA is therefore limited to its rate formation.

Regarding the elimination rate constant of 7-HCOU, it was graphically greater than the constant of COU. Ritschel and co-workers [32] proposed that 7-HCOU underwent an extensive first pass effect in the liver, leading to the formation of 7-hydroxycoumarin glucuronide. The first-pass effect of 7-HCOU might explain why the terminal slope of the plasma concentration curve resulted in a high value of K_el_ and why the area under the curve of 7-HCOU (126.0 ng*h/mL) was smaller than the areas of COU (1341.0 ng*h/mL) and OCA (465.0 ng*h/mL). In summary, the amounts of converted 7-HCOU in the liver were possibly high, but not all the 7-HCOU reached the systemic circulation because of its subsequent metabolism.

The calculated pharmacokinetic parameters showed a mean value of apparent clearance (Cl/f) of 1148.0 L/h for COU. Considering the bioavailability of COU (0.034 or 3.4%) calculated by Ritschel and co-workers [32], the estimated clearance in the present study was 39.03 L/h (Cl = 1148.0 x 0.034). This result showed the high capacity of organ clearance to eliminate COU, compared to the capacity to eliminate theophylline (2.8 L/h), a drug used as a reference in the treatment of asthma [40].

The calculated pharmacokinetic parameters also showed a mean value of apparent volume of distribution (Vd/f) of 6733.0 L for COU. Using the value of COU bioavailability (0.034 or 3.4%) calculated by Ritschel and co-workers [32], the volume of distribution was estimated at 228.9 L (Vd = 6733.0 x 0.034). Considering that a 70 kg individual has approximately 42.0 L of body fluid [41], a value of Vd of 228.9 L indicates that COU is amply distributed throughout the body.

The calculation of the pharmacokinetic parameters also demonstrated that the half-life (T_1/2_) of COU and metabolites was short (approximately 4.0 h for COU, 1.1 h for 7-HCOU and 3.5 h for OCA). Because T_1/2_ is a hybrid parameter of Cl and Vd (T_1/2_ = 0.632 x Vd/Cl) [42], it becomes evident that the short T_1/2_ was influenced by the capacity of the body to eliminate COU (elevated clearance).

Another relevant point is the low C_max_ of COU obtained with the 1500 mg dosage (735.0 ng/mL). Considering that the regulated daily intake of COU in some countries is only 5 mg (0.33% of the dose allometrically extrapolated to obtain a bronchodilator effect in humans), the conclusion is that the administration of guaco syrup under the recommended posology will not provide plasma concentrations of COU sufficient to promote a bronchodilator effect (at least not alone).

The same emphasis should be given to KAU, which promoted vasodilatory effects in rat aortas at concentrations greater than 10 mM (endothelium-intact or denuded rings) [10,11]. If administering 60 mL of guaco syrup (8.9 mg of the active metabolite) KAU was not detected in plasma volunteers, it is questionable whether the administration of the recommended dose (5 mL) would promote sufficient levels of the available KAU to have therapeutic effects.

Regarding the bronchodilator effects of 7-HCOU, it was obtained in groups of mice that orally received a 60 mg/kg dosage [18,29]. The 60 mg/kg dosage is equivalent to an allometric dose of 600 mg of pure 7-HCOU in a 70 kg individual. It is possible to conclude that with intake of guaco syrup, there is no possibility of obtaining sufficient levels of converted 7-HCOU to obtain a bronchodilation effect.

It is important to mention that the possible effects of guaco could be linked to the synergistic effects of its main metabolites. Therefore, studies that are more conclusive will be necessary to evaluate this possibility. Most importantly, the effectiveness of guaco syrup can only be determined by the conduction of clinical studies in humans. It is the key point for the development of safe and therapeutically useful preparations or to prevent public resources from being allocated for medicines that do not bring benefits to the population.

### 5. Identification of an alternative route of coumarin metabolism in humans

Coumarins are found at high levels in some essential oils, fruits, green tea, foods and medicinal plants [14]. The dietary exposure to coumarin is therefore quite significant in humans, and determining the metabolic fate of COU is important to access the possible dependence of coumarin-induced toxicity on metabolism.

The metabolism of COU has been investigated *in vitro* and *in vivo* in a wide range of species. COU can be converted into dihydrocoumarin, 7-hydroxycoumarin glucoronide, *o*-hydroxyphenylpropionic acid, 3, 4, 5, 6, 7 and 8-hydroxycoumarin, 6,7-dihydroxycoumarin, esculetin, *o*-hydroxyphenyllactic acid, *o*-hydroxyphenylacetic acid, *o*-hydroxyphenylacetaldehyde and *o*-hydroxyphenylethanol, however the biotransformation is specie-specific [14]. The conversion of COU into OCA has also been described, but only for rats and rabbits [14,17,22]. For humans, the majority of studies involving subjects have focused on 7-hydroxylation pathway.

7-HCOU has been considered the main metabolite of COU since 1950s. However, in this work the existence of a direct relationship between the amounts of available COU and the amounts of OCA in plasma volunteers was clearly evidenced (Fig. 2). More than that, the individual temporal profile of OCA demonstrated an AUC_0-t_ three times greater than that calculated for 7-HCOU (Table 5). This observation led to the conclusion that in addition to the 7-hydroxylation pathway (considered the major route), the hydrolysis of the lactone ring, forming a carboxylated compound, is one of the possible routes of COU metabolism in humans. This inedited result also demonstrated that OCA, more than 7-HCOU, is one of the main bioavailable metabolites of COU.

The formation of OCA in humans can be explained by the occurrence of chemical reactions in the intestine, where the basic medium provides favorable conditions to hydrolysis of the lactone ring [43]. The other possibility, which is more probable, is enzymatic hydrolysis of COU by the enzyme paraoxonase (PON1) present in human blood. The PON1 enzyme is an important catalyst of the hydrolysis of lactones, especially lactone rings containing from 4 to 7 atoms [44]. The simultaneous occurrence of chemical and enzymatic hydrolysis of COU cannot be excluded. It is important to mention that studies conducted in rats [43] and rat hepatocytes [45,46] demonstrated that the OCA may be a possible precursor of *o*-hydroxyphenylacetaldehyde and *o*-hydroxyphenylacetic acid, substances related to the liver damages in rodents. Therefore, the toxic and therapeutic effects of OCA in humans should be evaluated.

**Fig 4:**
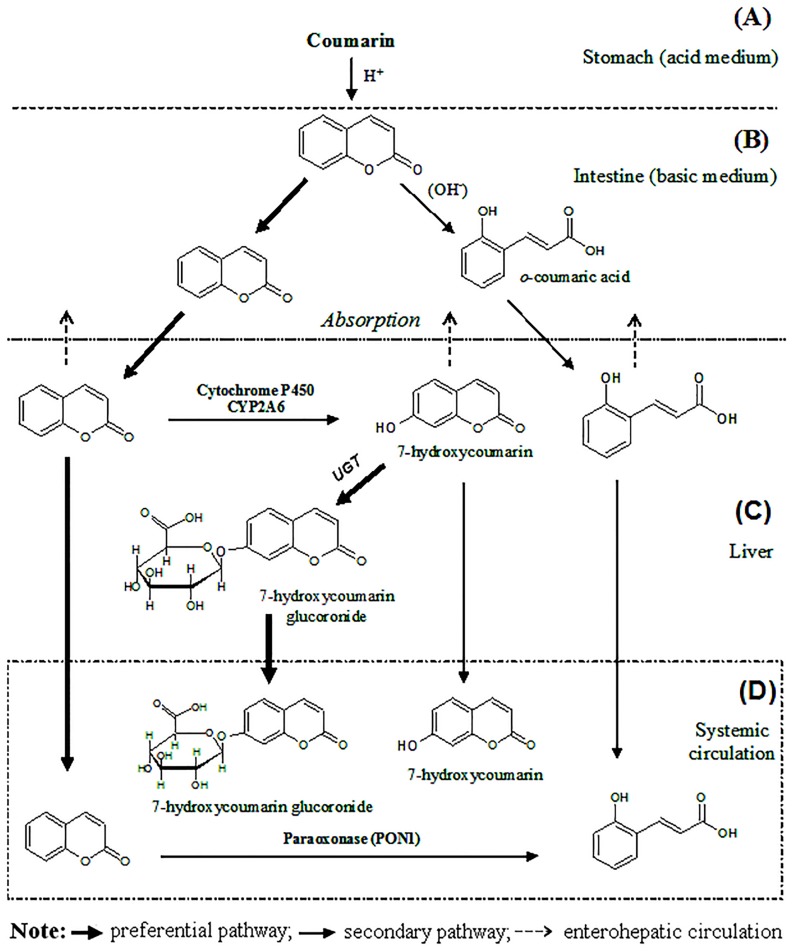
Metabolism of coumarin in humans via 7-hydroxylation and possible route via hydrolysis of the lactone ring.

Because significant levels of OCA were found in plasma volunteers, a route of COU metabolism in humans was proposed (Fig. 4). Note in Fig. 4A that the intaken coumarin arrives intact in the stomach due to the acid medium (pH: 1–2). Reaching the intestine (pH: 7–9), a fraction of coumarin remains intact, and another fraction can be hydrolysated to *o*-coumaric acid (Fig. 4B). Coumarin and converted *o*-coumarid acid are absorbed by the intestinal mucosa reaching the liver (Fig. 4C). A part of coumarin and *o*-coumaric acid reach the systemic circulation, and the fraction of coumarin that remained in the liver undergoes phase I metabolism by cytochrome CYP2A6, being converted to 7-hydroxycoumarin. A part of converted 7-hydroxycoumarin reaches the systemic circulation, and another part undergoes sequential metabolism, forming 7-hydroxycoumarin glucuronide by UDP-glucuronyltransferase (UGT). In the systemic circulation (Fig. 4D), coumarin undergoes enzymatic hydrolysis by the enzyme paraoxonase (PON1) being converted to *o*-coumaric acid. The proposed route of coumarin metabolism in humans via 7-hydroxylation and possible route via hydrolysis of the lactone ring is shown in Fig. 4.

## Conclusion

The new, reproducible, sensitive, fast and fully validated HPLC-MS/MS methods developed in this study were found to be suitable for the quantification of COU, 7-HCOU, OCA and KAU in human plasma. The methods were selective, linear, precise, accurate and free of matrix effects. Excellent stability for all the compounds was also shown.

The administration of 60 mL of guaco syrup (single dose) demonstrated that the amounts of available COU, 7-HCOU, OCA and KAU were insufficient to be detected by high-sensitivity methods. The kinetic study, conducted using 60 mL of guaco syrup spiked with 1500 mg of COU, resulted in significant plasma levels of COU and its metabolites (7-HCOU and OCA). The study demonstrated that COU had low bioavailability, and the formation of its converted metabolites was fully dependent of COU dosage. The kinetic study also allowed for the observation that OCA is one of the main metabolites of COU *in vivo* and that the elimination of OCA is dependent of its rate of formation. The plasma levels of 7-HCOU were extremely low, and its kinetic profile suggested sequential hepatic metabolism with low access to the systemic circulation.

The present study showed that the administration of guaco syrup under the recommended posology would not provide plasma concentrations of COU sufficient to promote a bronchodilator effect (at least not alone). Therefore, the possible synergistic effects among COU, 7-HCOU, OCA and KAU should be evaluated to understand the true mechanism of action of guaco. Most importantly, clinical studies evaluating the effectiveness of guaco syrup are necessary to guarantee benefits for the population or to avoid government spending.

## Supporting Information

S1 Fig Identification of kaurenoic acid isolated from *M*. *lanuginosa* DC (IR, GC/MS, 1H and 13C NMR). (TIF)

S2 Fig Chromatograms obtained by HPLC-MS/MS for the selectivity study. Positive ion mode: (a) normal blank plasma; (b) lipemic blank plasma; (c) hemolyzed blank plasma; (d) normal blank plasma spiked with coumarin (COU), 7-hydroxycoumarin (7-HCOU) and IS 6-methylcoumarin (6-MC) at LLOQ concentrations.
(TIF)

S3 Fig Chromatograms obtained by HPLC-MS/MS for the selectivity study. Negative ion mode: (a) normal blank plasma; (b) lipemic blank plasma; (c) hemolyzed blank plasma; (d) normal blank plasma spiked with *o*-coumaric acid (OCA), kaurenoic acid (KAU) and ISs isoferulic acid (ISOF) and prednisone (PRED) at LLOQ concentrations.
(TIF)

S1 Table Stability data for coumarin, 7-hydroxycoumarin and 6-methylcoumarin (IS) under various storage conditions (n = 8). (DOCX)

S2 Table Stability data for *o*-coumaric acid, kaurenoic acid, isoferulic acid (IS) and prednisone (IS) under various storage conditions (n = 8). (DOCX)
