# Supplementary figures and images for: A Kinetic Study of the Main Guaco Metabolites Using Syrup Formulation and the Identification of an Alternative Route of Coumarin Metabolism in Humans
Source: PLoS One. 2015 Mar 10;10(3):e0118922. doi: 10.1371/journal.pone.0118922 (PMC4355590; doi:10.1371/journal.pone.0118922)

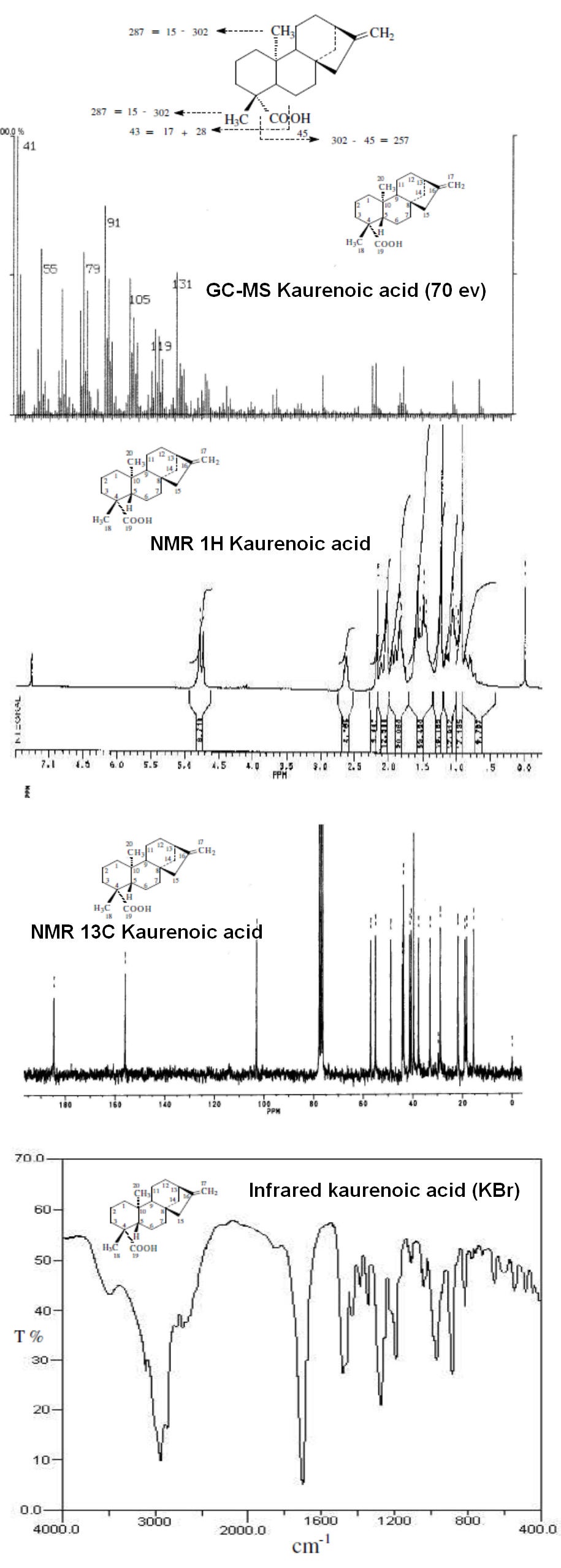

Supplement: S1 Fig — (TIF) [file pone.0118922.s001.tif]

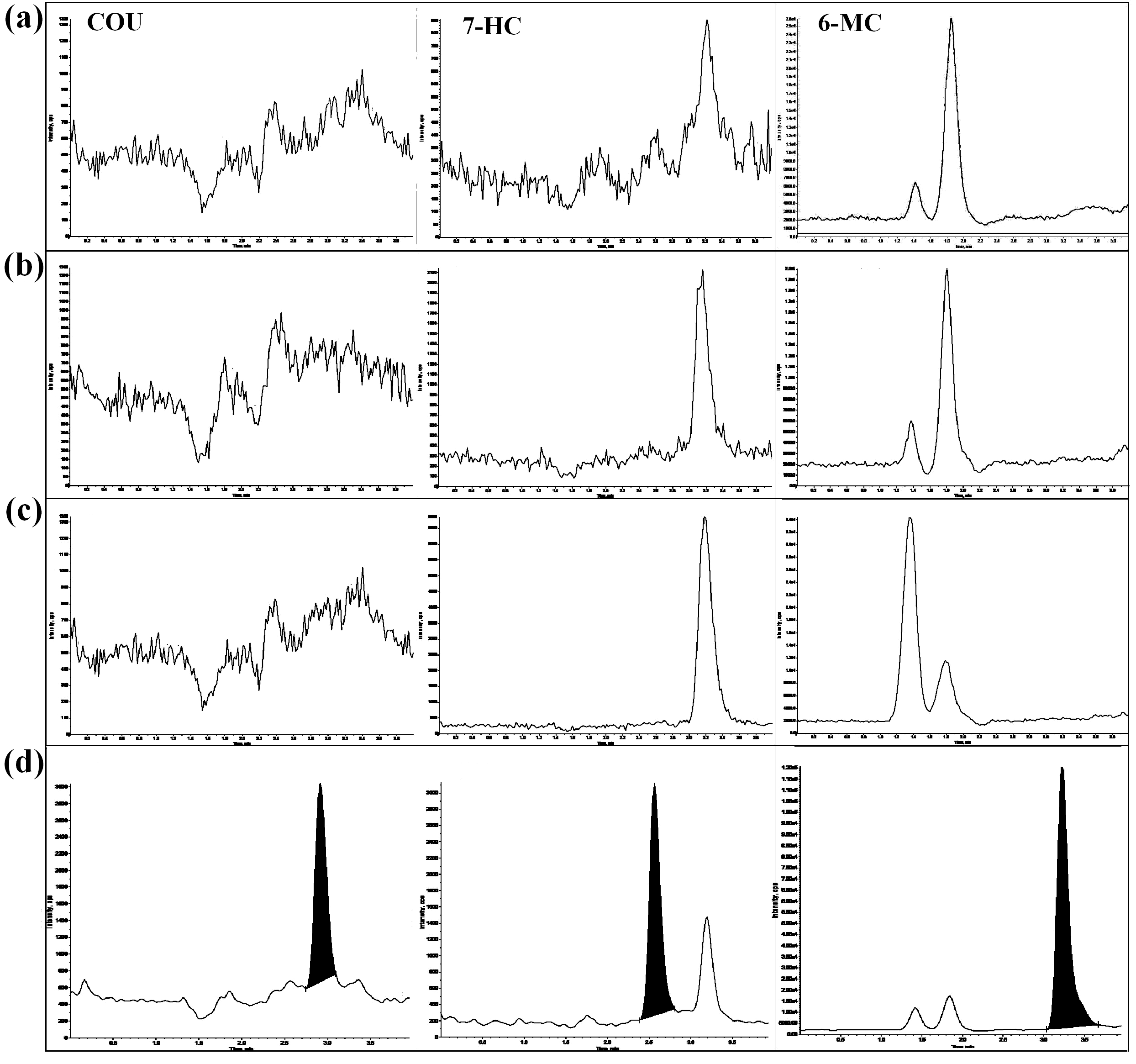

Supplement: S2 Fig — Positive ion mode: (a) normal blank plasma; (b) lipemic blank plasma; (c) hemolyzed blank plasma; (d) normal blank plasma spiked with coumarin (COU), 7-hydroxycoumarin (7-HCOU) and IS 6-methylcoumarin (6-MC) at LLOQ concentrations. (TIF) [file pone.0118922.s002.tif]

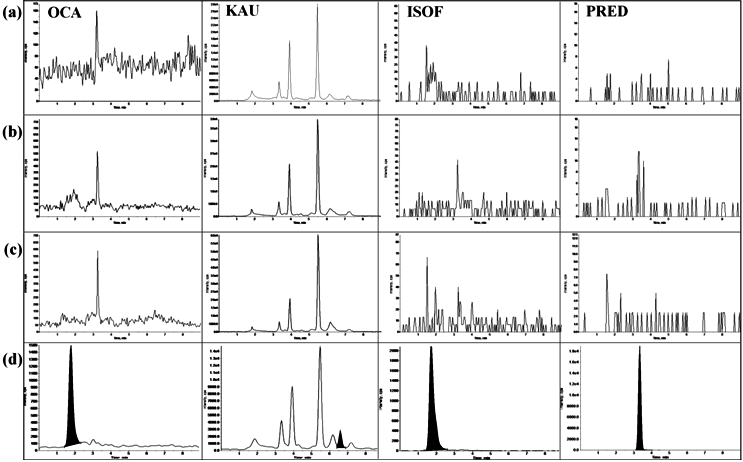

Supplement: S3 Fig — Negative ion mode: (a) normal blank plasma; (b) lipemic blank plasma; (c) hemolyzed blank plasma; (d) normal blank plasma spiked with o-coumaric acid (OCA), kaurenoic acid (KAU) and ISs isoferulic acid (ISOF) and prednisone (PRED) at LLOQ concentrations. (TIF) [file pone.0118922.s003.tif]
