# Supplementary material for: A Kinetic Study of the Main Guaco Metabolites Using Syrup Formulation and the Identification of an Alternative Route of Coumarin Metabolism in Humans
Source: PLoS One. 2015 Mar 10;10(3):e0118922. doi: 10.1371/journal.pone.0118922 (PMC4355590; doi:10.1371/journal.pone.0118922)
Supplement: S1 Table — (DOCX) [file pone.0118922.s004.docx]

**Table S1**. Stability data for coumarin, 7-hydroxycoumarin and 6-methylcoumarin (IS) under various storage conditions (n = 8)

| **Stability** | | |  | **Coumarin** | | |  | **7-hydroxycoumarin** | | |  | **6-methylcoumarin*** |
| --- | --- | --- | --- | --- | --- | --- | --- | --- | --- | --- | --- | --- |
|  |  |  |  | Level 50.0 |  | Level 1250.0 |  | Level 12.5 |  | Level 750.0 |  | Level 500.0 |
|  |  |  |  | (ng/mL) |  | (ng/mL) |  | (ng/mL) |  | (ng/mL) |  | (ng/mL) |
| **8 h at room** | **temperature** | Mean recovery (ng/mL ± SD) |  | 54.5 ± 3.2 |  | 1293.1 ± 30.5 |  | 11.6 ± 0.6 |  | 757.9 ± 5.6 |  | 497.3 ± 3.8 |
|  |  | RSD (%) |  | 5.81 |  | 2.36 |  | 5.66 |  | 0.74 |  | 0.76 |
|  |  | RE (%) |  | 8.94 |  | 3.45 |  | -7.41 |  | 1.06 |  | -0.54 |
|  | |  |  |  |  |  |  |  |  |  |  |  |
| **30 days** | **at 4 °C** | Mean recovery (ng/mL ± SD) |  | 56.1 ± 2.3 |  | 1173.4 ± 67.9 |  | 11.4 ± 0.9 |  | 791.8 ± 60.5 |  | 480.6 ± 46.6 |
|  |  | RSD (%) |  | 4.04 |  | 5.78 |  | 7.59 |  | 7.64 |  | 9.69 |
|  |  | RE (%) |  | 12.27 |  | -6.13 |  | -8.02 |  | 5.57 |  | -3.89 |
|  | |  |  |  |  |  |  |  |  |  |  |  |
| **Short-ter** |  | Mean recovery (ng/mL ± SD) |  | 52.4 ± 1.7 |  | 1205.5 ± 31.5 |  | 11.7 ± 0.6 |  | 738.1 ± 8.4 |  | 501.1 ± 1.6 |
|  |  | RSD (%) |  | 3.25 |  | 2.61 |  | 4.71 |  | 1.14 |  | 0.32 |
|  |  | RE (%) |  | 4.81 |  | -3.56 |  | -6.24 |  | -1.59 |  | 0.45 |
|  | |  |  |  |  |  |  |  |  |  |  |  |
| **Freeze–thaw** | **cycles** | Mean recovery (ng/mL ± SD) |  | 53.8 ± 2.7 |  | 1307.7 ± 40.7 |  | 11.6 ± 0.6 |  | 714.8 ± 24.9 |  | NA |
|  |  | RSD (%) |  | 5.01 |  | 3.11 |  | 5.56 |  | 3.48 |  | NA |
|  |  | RE (%) |  | 7.62 |  | 4.61 |  | -7.29 |  | -4.69 |  | NA |
|  | |  |  |  |  |  |  |  |  |  |  |  |
| **Long** | **term** | Mean recovery (ng/mL ± SD) |  | 45.1 ± 3.6 |  | 1265.7 ± 99.2 |  | 11.7 ± 1.1 |  | 674.4 ± 69.7 |  | NA |
|  |  | RSD (%) |  | 7.90 |  | 7.84 |  | 9.34 |  | 10.33 |  | NA |
|  |  | RE (%) |  | -9.71 |  | 1.26 |  | -6.19 |  | -10.08 |  | NA |
|  | |  |  |  |  |  |  |  |  |  |  |  |
| **post-** | **preparative** | Mean recovery (ng/mL ± SD) |  | 47.6 ± 3.4 |  | 1281.3 ± 44.2 |  | 11.5 ± 0.7 |  | 776.4 ± 18.7 |  | 468.1 ± 22.6 |
|  |  | RSD (%) |  | 7.09 |  | 3.45 |  | 5.93 |  | 2.40 |  | 4.66 |
|  |  | RE (%) |  | -9.55 |  | 5.00 |  | -7.73 |  | 3.52 |  | -6.38 |

NA: not applicable; *internal standard
