# Supplementary material for: A Kinetic Study of the Main Guaco Metabolites Using Syrup Formulation and the Identification of an Alternative Route of Coumarin Metabolism in Humans
Source: PLoS One. 2015 Mar 10;10(3):e0118922. doi: 10.1371/journal.pone.0118922 (PMC4355590; doi:10.1371/journal.pone.0118922)
Supplement: S2 Table — (DOCX) [file pone.0118922.s005.docx]

**Table S2**. Stability data for *o*-coumaric acid, kaurenoic acid, isoferulic acid (IS) and prednisone (IS) under various storage conditions (n = 8)

| **Stability** | | |  | ***o*-coumaric acid** | | |  | **Kaurenoic acid** | | |  | | **Isoferulic acid*** |  | **Prednisone*** |  |
| --- | --- | --- | --- | --- | --- | --- | --- | --- | --- | --- | --- | --- | --- | --- | --- | --- |
|  |  |  |  | Level 25.0 (ng/mL) |  | Level 750.0 (ng/mL) |  | Level 10.0 (ng/mL) |  | Level 500.0 (ng/mL) |  | | Level 1000.0 (ng/mL) |  | Level 500.0 (ng/mL) |  |
| **8 h at room** | **temperature** | Mean recovery (ng/mL ± SD) |  | 22.6 ± 1.3 |  | 718.0 ± 48.0 |  | 10.2 ± 0.7 |  | 516.6 ± 5.1 |  | | 950.0 ± 75.2 |  | 470.5 ± 26.6 |  |
|  |  | RSD (%) |  | 7.00 |  | 3.08 |  | 6.05 |  | 2.32 |  | | 3.63 |  | 4.30 |  |
|  |  | RE (%) |  | -9.44 |  | -4.26 |  | 8.94 |  | 3.33 |  | | -5.00 |  | -5.90 |  |
|  | |  |  |  |  |  |  |  |  |  |  | |  |  |  |  |
| **30 days** | **at 4 °C** | Mean recovery (ng/mL ± SD) |  | 22.8 ± 1.2 |  | 640.8 ± 45.6 |  | 9.75 ± 0.6 |  | 487.8 ± 34.9 |  | | 1037.5 ± 68.1 |  | 497.0 ± 13.9 |  |
|  |  | RSD (%) |  | 6.59 |  | 11.09 |  | 1.76 |  | 2.32 |  | | 2.60 |  | 4.30 |  |
|  |  | RE (%) |  | -8.89 |  | -14.55 |  | -2.46 |  | 3.33 |  | | 3.75 |  | -5.90 |  |
|  | |  |  |  |  |  |  |  |  |  |  | |  |  |  |  |
| **Short-term** |  | Mean recovery (ng/mL ± SD) |  | 25.2 ± 0.4 |  | 750.6 ± 29.0 |  | 10.0 ± 0.3 |  | 498.9 ± 7.6 |  | | 999.3 ± 62.2 |  | 502.2 ± 11.1 |  |
|  |  | RSD (%) |  | 0.56 |  | 0.05 |  | 0.16 |  | 0.15 |  | | 0.04 |  | 0.31 |  |
|  |  | RE (%) |  | 0.79 |  | 0.07 |  | 0.23 |  | -0.22 |  | | -0.07 |  | 0.43 |  |
|  | |  |  |  |  |  |  |  |  |  |  |  | |  |  | |
| **Freeze–thaw** | **cycles** | Mean recovery (ng/mL ± SD) |  | 22.9 ± 1.1 |  | 729.0 ± 41.7 |  | 9.9 ± 0.4 |  | 502.5 ± 6.1 |  | NA | |  | NA | |
|  |  | RSD (%) |  | 6.14 |  | 2.00 |  | 0.29 |  | 0.35 |  | NA | |  | NA | |
|  |  | RE (%) |  | -8.32 |  | -2.79 |  | -0.41 |  | 0.49 |  | NA | |  | NA | |
|  | |  |  |  |  |  |  |  |  |  |  |  | |  |  | |
| **Long-term** |  | Mean recovery (ng/mL ± SD) |  | 22.5 ± 0.4 |  | 606.3 ± 26.8 |  | 8.8 ± 0.3 |  | 463.2 ± 12.3 |  | NA | |  | NA | |
|  |  | RSD (%) |  | 7.60 |  | 5.25 |  | 8.79 |  | 5.41 |  | NA | |  | NA | |
|  |  | RE (%) |  | -10.20 |  | -7.16 |  | -11.70 |  | -7.36 |  | NA | |  | NA | |
|  | |  |  |  |  |  |  |  |  |  |  |  | |  |  | |
| **post-** | **preparative** | Mean recovery (ng/mL ± SD) |  | 22.7 ± 0.6 |  | 727.4 ± 34.5 |  | 9.8 ± 0.4 |  | 509.8 ± 6.6 |  | 986.5 ± 31.7 | |  | 479.3 ± 16.9 | |
|  |  | RSD (%) |  | 1.66 |  | 2.16 |  | 1.21 |  | 1.37 |  | 0.96 | |  | 2.99 | |
|  |  | RE (%) |  | -2.32 |  | -3.01 |  | -1.70 |  | 1.96 |  | -1.35 | |  | -4.14 | |

NA: not applicable; *internal standard
